# Explantation, Guided Bone Regeneration, and Digitally Planned Implant Replacement in Advanced Peri-Implantitis: A Case Report With 12-Month Follow-Up

**DOI:** 10.7759/cureus.117506

**Published:** 2026-10-05

**Authors:** Adriana María Sánchez Calvo, Sarah Ocaña Chacón, Daniela Carmona Ruiz, César Esquivel Chirino

**Affiliations:** 1 Department of Periodontics, Universidad Intercontinental, Mexico City, MEX; 2 Department of Orthodontics, Faculty of Dentistry, Universidad Nacional Autónoma de México, Mexico City, MEX; 3 Department of Basic Medical Sciences, Faculty of Dentistry, Universidad Nacional Autónoma de México, Mexico City, MEX

**Keywords:** dental implant explantation, guided bone regeneration, implantoplasty, periimplantitis, static guided surgery

## Abstract

When advanced peri-implantitis affects several implants, it is necessary to decide which implants to retain and which to remove before planning any reconstruction. This case highlights the indications for explantation in advanced peri-implantitis, the role of guided bone regeneration in reconstructing compromised sites, and the benefits of digitally planned placement of new implants. The 12-month follow-up underscores the clinical relevance of sustained peri-implant health.

We present the clinical case of a 65-year-old man with arterial hypertension and type 2 diabetes mellitus under treatment (glycated hemoglobin 6% at baseline, non-smoker) who presented with a fracture of his upper fixed prosthesis and esthetic concerns. Generalized stage IV grade B periodontitis was diagnosed, along with peri-implantitis in the seven existing implants, whose defects were classified on cone beam computed tomography between classes Ib and IIIc. Six implants were assigned a hopeless prognosis, and one was given a guarded prognosis. After initial periodontal and prosthetic therapy, six implants were removed, with forceps in the maxilla and with a trephine followed by forceps in the mandible, and the root remnants were extracted. The retained implant was treated by implantoplasty and surface decontamination. The explantation sites were regenerated with allograft and a resorbable collagen membrane. Four months later, 12 implants were placed with static guided surgery, using a mucosa-supported guide in the maxilla and two bone-supported guides for trimming and placement in the mandible, all achieving an insertion torque above 35 N·cm. At position 44, plasma rich in growth factors mixed with autologous bone was added. Rehabilitation was completed with screw-retained fixed prostheses in both arches. At 12 months after delivery of the definitive prostheses, the probing depth remained between 3 and 4 mm in all the implants, with no bleeding on probing and no evident radiographic progression of marginal bone loss compared to the initial record. This case demonstrates that a stepwise sequence of selective explantation, regeneration, and guided placement of new implants is feasible in advanced peri-implantitis; however, as a single observation in a well-controlled, non-smoking patient, it demonstrates short-term clinical feasibility only and cannot establish effectiveness, predictability, or reproducibility.

## Introduction

Peri-implantitis is an inflammatory lesion associated with biofilms that affects the tissues surrounding the implant and is accompanied by progressive loss of the supporting bone [1]. Its frequency varies among studies; in the systematic review by Derks and Tomasi, the weighted prevalence at the patient level was 43% for peri-implant mucositis and 22% for peri-implantitis [2].

Among the associated factors, the evidence is most consistent for a history of periodontitis, poor plaque control, and lack of periodic maintenance; for smoking and diabetes, the available data are less conclusive [1]. The morphology of the defect also influences treatment options; accordingly, Monje et al. proposed a classification based on the pattern and severity of bone loss assessed with cone beam computed tomography [3].

When an implant has a reasonable prognosis, surgical therapy may include debridement, surface decontamination, and implantoplasty [4]. The decision on whether an implant is salvageable or hopeless is based not on a single parameter but on the combination of defect configuration and severity, probing depth, bleeding on probing and suppuration, implant mobility, the amount of residual supporting bone, implant position, cleansability, and prosthetic value. If bone loss irreversibly compromises the support, explantation becomes the alternative, and reconstruction of the defect is the next step to allow for new rehabilitation [5,6]. Explantation is generally indicated when the implant is mobile, when peri-implant bone loss is advanced and circumferential, when the defect morphology precludes reconstructive therapy with a predictable outcome, or when repeated non-surgical and surgical therapy has failed to arrest disease progression [3,5].

Guided bone regeneration (GBR) then relies on well-established biological principles: appropriate barrier-membrane selection, space maintenance and graft stability, and tension-free primary closure to restore the bone volume needed to place the new implants in a prosthetically driven position rather than leaving the residual defect to dictate the final rehabilitation [7].

Regarding the placement of new implants in previously regenerated sites, three-dimensional digital planning and surgical guides offer alternatives to freehand placement. The available reviews describe deviations within clinically manageable ranges, although they emphasize maintaining a safety margin from anatomical structures and warn about procedure-specific complications [8,9]. Digitally planned placement allows the new implants to be positioned according to the reconstructed anatomy and the planned prosthesis, improves reproducibility over freehand placement, and, in compromised sites, helps to avoid the grafted areas and adjacent structures [8,9]. Beyond positional accuracy, computer-assisted planning has been associated with more predictable, prosthetically driven outcomes in complex reconstructions, which is particularly relevant when implants must be placed in previously regenerated bone, where the anatomical references are altered and the margin for error is reduced [8,9].

Finally, because peri-implant tissues can relapse over time, a follow-up of at least 12 months is relevant to document that the treated and newly placed implants remain stable, with maintained probing depths, absence of bleeding, and no further radiographic progression of marginal bone loss [1].

The individual procedures used here (implantoplasty, explantation, GBR, and guided implant placement) are all well established. The educational contribution of this report is not a new technique but the integration of these steps into a single, staged, full-mouth treatment sequence for a patient with peri-implantitis affecting all of his implants. This case report describes the clinical management of a patient with generalized peri-implantitis affecting all seven implants (six judged hopeless and one retained and treated), which was resolved through selective explantation, guided bone regeneration, and digitally planned implant replacement, with a 12-month follow-up after definitive rehabilitation.

## Case presentation

A 65-year-old man visited the Periodontics postgraduate clinic at the Intercontinental University because of a fractured upper prosthesis and dissatisfaction with his appearance. His medical history included controlled arterial hypertension and type 2 diabetes mellitus (metformin; most recent glycated hemoglobin of 6%). On examination, supragingival biofilm and calculus were found around the teeth, implants, and prostheses, with generalized edema, erythema, and bleeding on probing. The gingival phenotype was thick. The upper fixed prosthesis was fractured and ill-fitting, and screw-retained single crowns had been placed on the implants. The alveolar ridge corresponded to Seibert Class III in the anteroinferior sector and Class II in the posteroinferior sector. The patient retained several natural teeth and was therefore not completely edentulous at the start of treatment (Figure 1). Interproximal clinical attachment loss exceeded 5 mm at multiple sites, with more than 20 teeth lost to periodontitis. Maximum probing depths reached 6-8 mm in several teeth, accompanied by grade II-III mobility. Radiographic bone loss was approximately 50% of root length; given the patient's age of 65 years, the bone-loss-to-age ratio was approximately 0.77, consistent with grade B progression. The baseline plaque index was 60-70%, and bleeding on probing was detected at more than 50% of sites, confirming generalized inflammation. 

**Figure 1:**
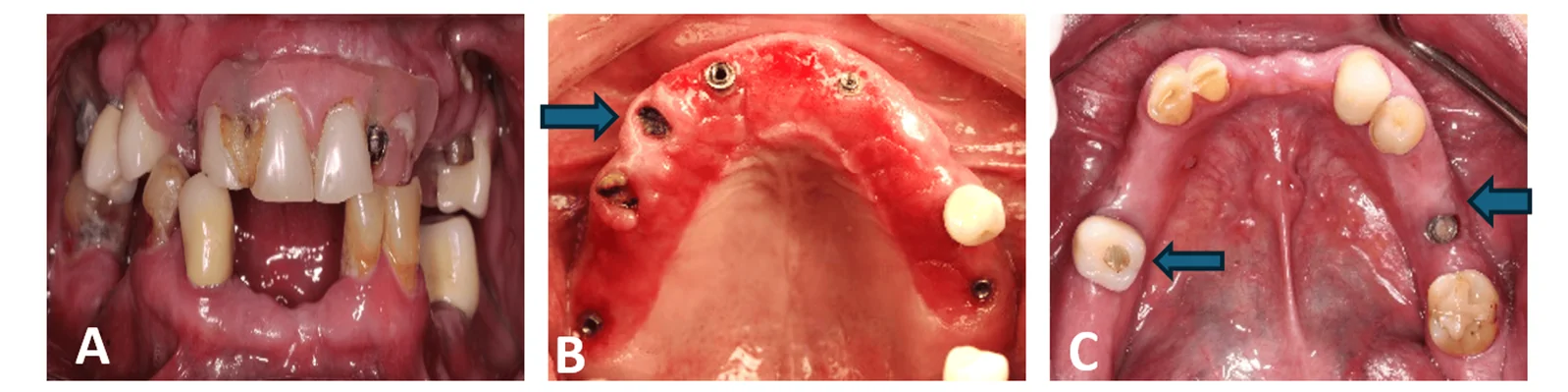
Clinical diagnosis. (A) Initial intraoral view showing edema, erythema, bleeding on probing, a fractured upper fixed prosthesis, and supragingival biofilm. (B) Upper sector with root remnants and caries after removal of the prosthesis. (C) Lower arch with an unrestored implant, a crown on an implant, and edentulous areas.

Cone beam computed tomography revealed bone loss around the seven implants, and a diagnosis of generalized stage IV grade B periodontitis with peri-implantitis affecting all the implants was established. The defects were classified according to Monje et al. [3] as follows: 17, class Ib; 15, class IIIb; 12, class II; 22, class Ib; 27, class IIIb; 36, class IIIc; and 46, class IIIc (Figure 2). The extent of bone loss led to a hopeless prognosis for six implants and for the remaining teeth and a guarded prognosis for the implant in position 17.

**Figure 2:**
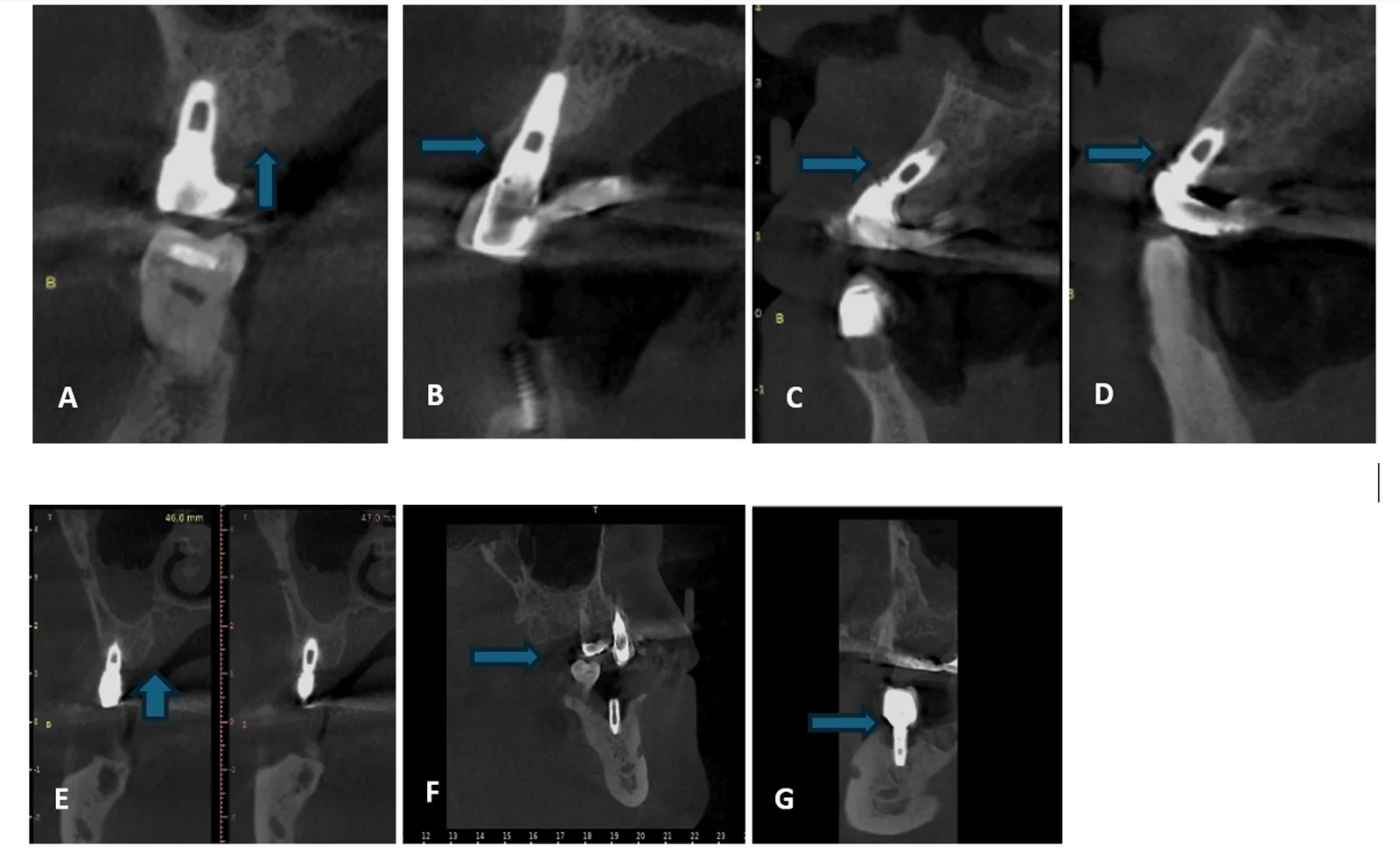
Radiographic diagnosis using cone beam computed tomography. Transverse sections of peri-implant defects. (A) Position 17, class Ib; (B) 15, class IIIb; (C) 12, class II; (D) 22, class Ib; (E) 27, class IIIb; (F) 36, class IIIc; (G) 46, class IIIc.

Implants 17 and 22 shared the same type of defect; therefore, the decision to preserve the former and remove the latter was based on the magnitude of bone loss, the probing depth (which reached 6 mm at implant 22), and the presence of bleeding on probing. A full-mouth, six-site periodontal and peri-implant chart was recorded at baseline, documenting probing depth, gingival margin, plaque, and bleeding on probing for all teeth and implants; none of the implants showed suppuration or clinical mobility (Figure 3). The baseline peri-implant status of each implant, the treatment decision, and the 12-month outcome are summarized in Table 1. 

**Table 1:** Baseline peri-implant status, treatment decision, and 12-month outcome. The defect class is according to Monje et al. [3]. Baseline values are from the initial examination; 12-month values correspond to the retained implant (17) and the newly placed implants. PD: probing depth, BoP: bleeding on probing.

Site (FDI)	Defect class	Baseline PD (mm)	Baseline BoP	Buccal keratinized mucosa (mm)	Prognosis	Decision	12-month PD (mm)	12-month BoP
17	Ib	5	NO	2	Guarded	Retained + implantoplasty	3-4	No
15	IIIb	7	YES	1	Hopeless	Explanted + GBR	-	-
12	II	6	YES	1	Hopeless	Explanted + GBR	-	-
22	Ib	6	Yes	1	Hopeless	Explanted + GBR	-	-
27	IIIb	8	YES	1	Hopeless	Explanted + GBR	-	-
36	IIIc	7	YES	1	Hopeless	Explanted + GBR	-	-
46	IIIc	8	YES	1	Hopeless	Explanted + GBR	-	-
6 maxillary implants (new)	-	-	-		-	Placed (guided)	3-4	No
6 mandibular implants (new)	-	-	-		-	Placed (guided)	3-4	No

**Figure 3:**
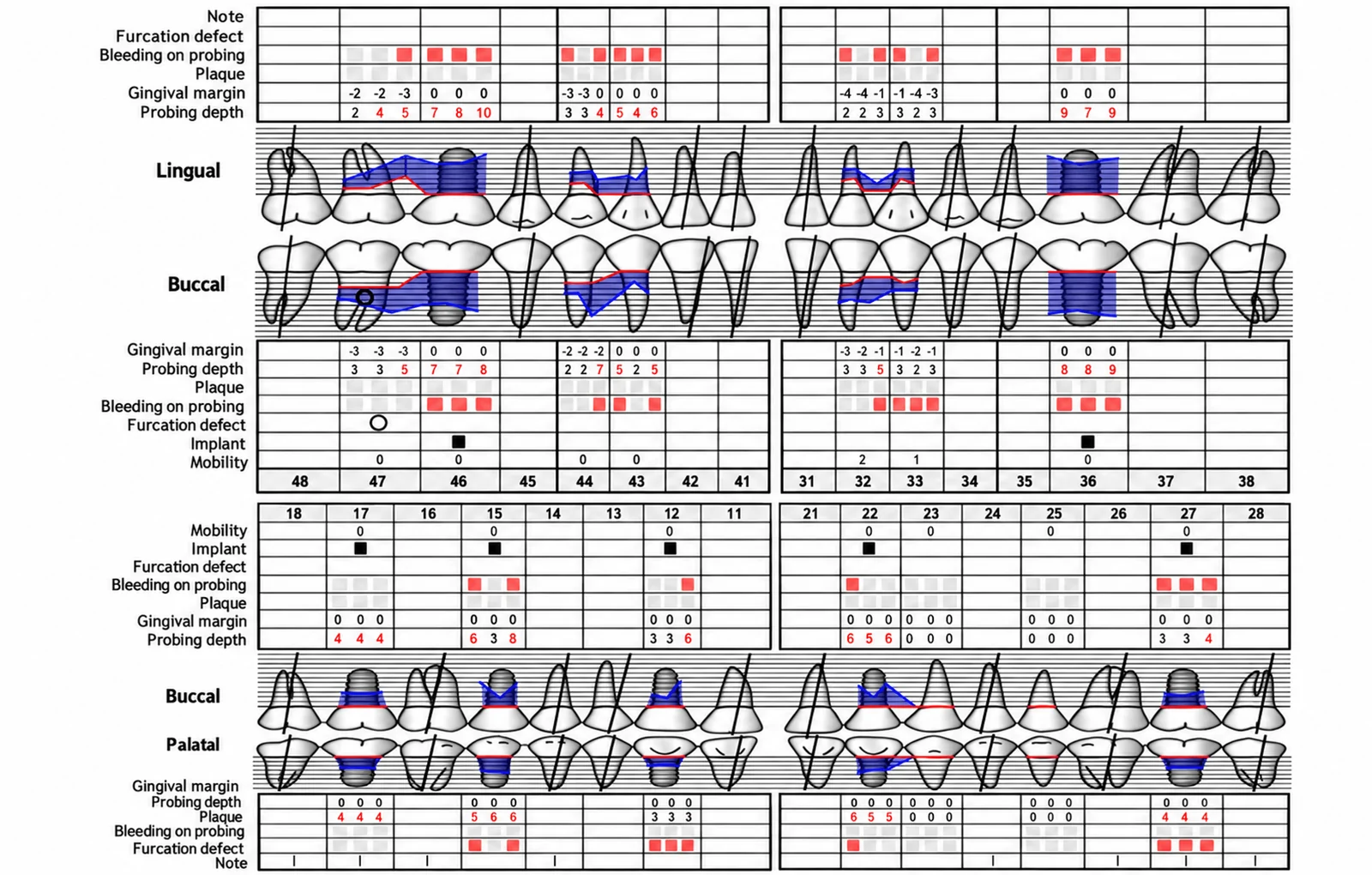
Baseline periodontal and peri-implant charting (periodontogram). Six-site recordings of probing depth, gingival margin, plaque, and bleeding on probing are shown for the maxillary and mandibular teeth and implants, together with furcation involvement and mobility. At baseline, none of the implants exhibited suppuration or clinical mobility; the absence of purulent exudate and the stability of all fixtures were confirmed during probing and functional testing, supporting a diagnosis of peri-implantitis based primarily on probing depth, bleeding on probing, and radiographic bone loss rather than implant mobility.

The plan comprised an initial periodontal and prosthetic phase (scaling and root planing in all four quadrants, reinforcement of oral hygiene techniques, and provision of a diagnostic wax-up and provisional prostheses) and a surgical phase that included the explantation of six implants, regeneration of the resulting beds, resective treatment of the preserved implant, and, later, the placement of six implants in the maxilla and six in the mandible, with definitive rehabilitation using screw-retained fixed prostheses.

In the first surgical intervention, the six hopeless implants were explanted and their sites were prepared for regeneration. The maxilla was anesthetized by regional infiltration with 4% articaine and 1:100,000 epinephrine, and the mandible was blocked with a bilateral inferior alveolar nerve block. In the maxilla, four implants with advanced horizontal bone loss were removed using the forceps technique, and two root remnants were extracted from the second quadrant. In the mandible, two internal-connection implants were removed with a 5.1 mm trephine followed by forceps because the density of the mandibular bone did not allow their removal with forceps alone (Figure 4) [5,10].

**Figure 4:**
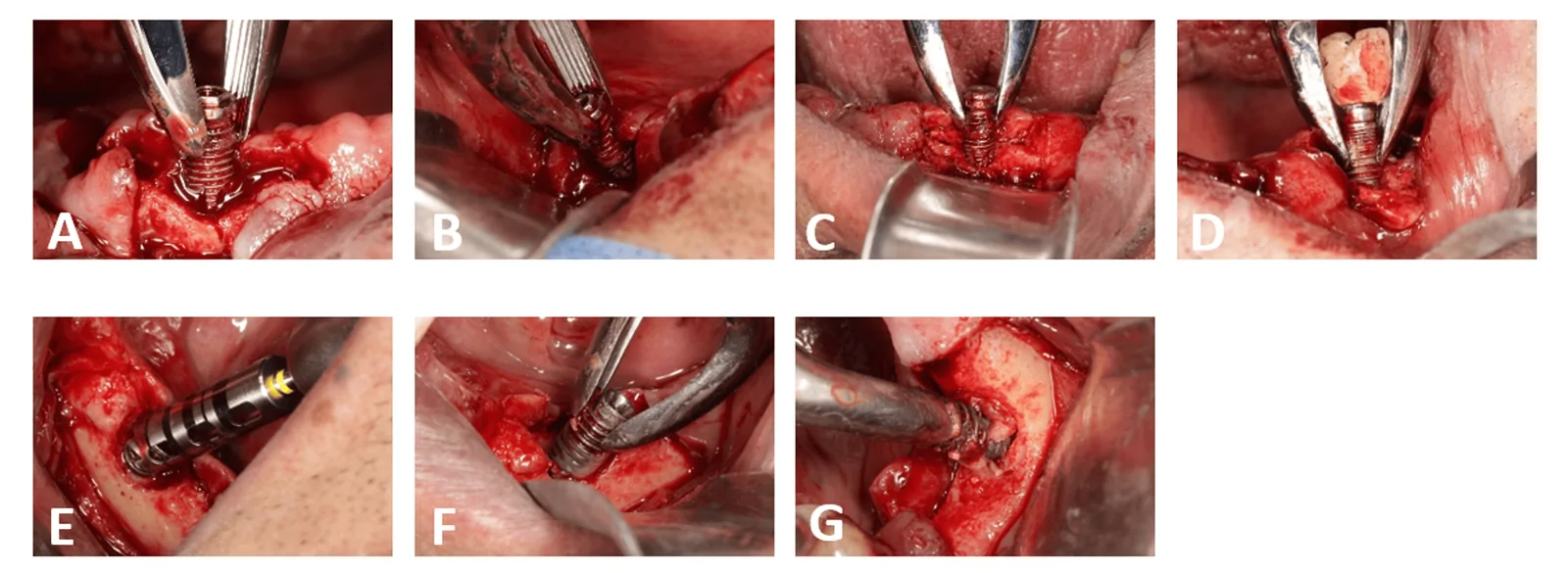
Explantations. (A) Implant 12 with forceps; (B) implant 27 with forceps; (C) implant 22 with forceps; (D) implant 15 with forceps; (E-G) mandibular implants removed with a 5.1 mm trephine followed by forceps, preserving the remaining bone contour.

The implant at position 17, which showed mild horizontal bone loss, was preserved and underwent resective treatment following implantoplasty and decontamination [4]. A full-thickness flap was raised to expose the threads, and implantoplasty was performed with a ball-shaped diamond bur, protecting the field with gauze to contain loose particles; the threads were then polished with metal-specific rubber. Granulation tissue was removed with ultrasound and steel curettes, a titanium brush was passed over the surface, and 5% hydrogen peroxide was applied for one minute. Suturing was performed with horizontal mattress sutures and simple 5-0 nylon sutures (Figure 5).

**Figure 5:**
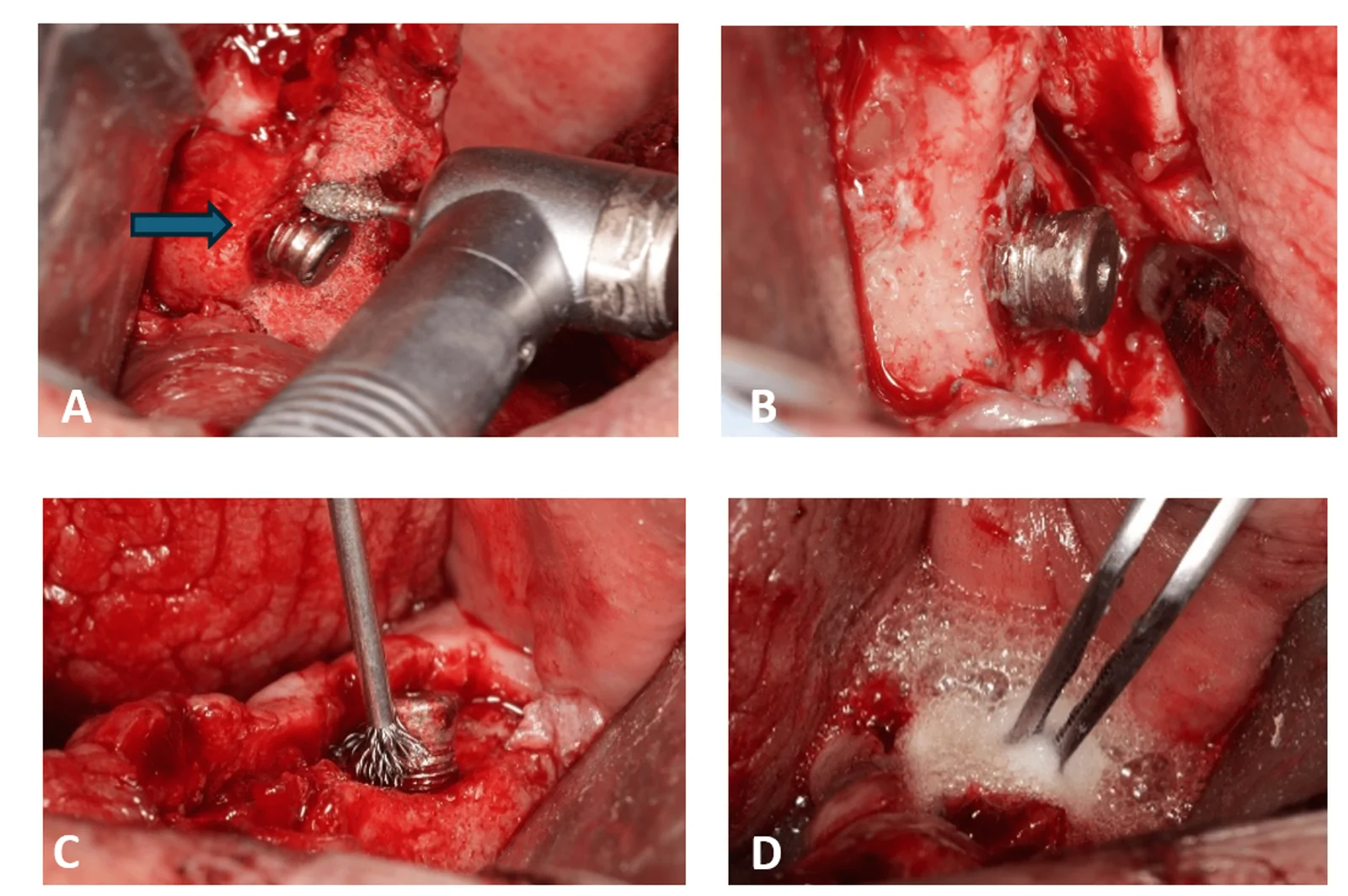
Resective treatment of peri-implantitis on implant 17. (A) Full-thickness flap exposing the threads before implantoplasty; (B) surface polishing with a diamond bur and debris control with gauze; (C-D) titanium brush and application of 5% hydrogen peroxide for decontamination.

The explantation beds in all four quadrants were regenerated with allograft (MinerOss, BioHorizons, Birmingham, Alabama) and a resorbable collagen membrane (Mem-Lok, BioHorizons, Birmingham, Alabama; a type I bovine collagen matrix with a resorption time of 26 to 38 weeks), fixed to the periosteum using the sling technique with 5-0 polyglactin resorbable suture. Corticotomies were also performed in the fourth quadrant to promote regeneration. The flap was released and closed with horizontal mattress sutures and simple 5-0 nylon sutures (Figure 6) [6,7].

**Figure 6:**
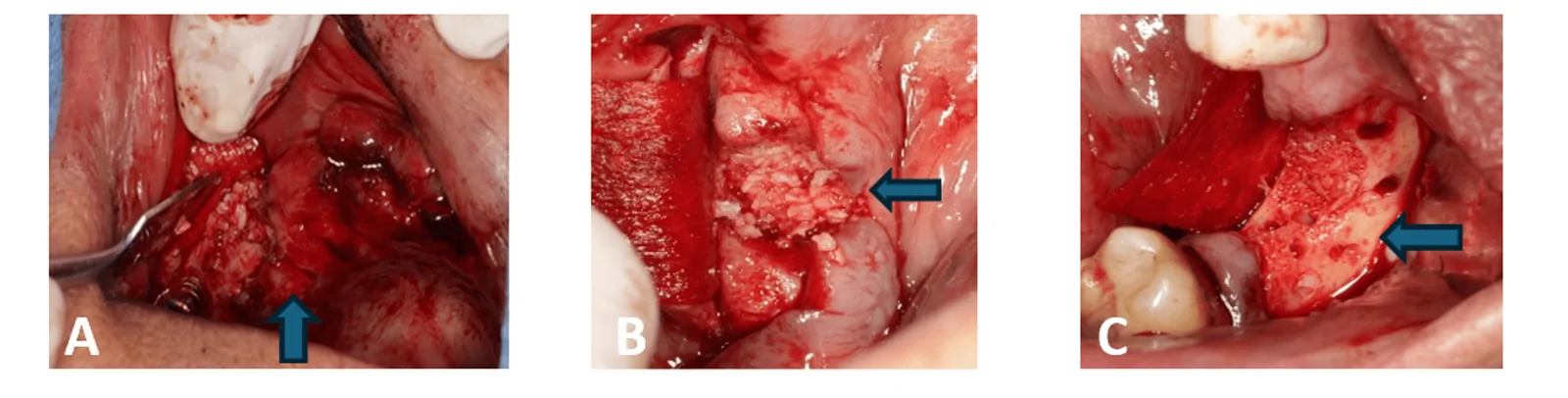
Guided bone regeneration. Representative quadrants are shown: (A) quadrant I, allograft and resorbable membrane fixed with the sling technique in the explantation area; (B) quadrant III, allograft and resorbable membrane in the explantation area; (C) quadrant IV, allograft and resorbable membrane in the explantation area, with corticotomies to promote regeneration. The second quadrant was regenerated with the same protocol.

After four months of healing, a new full-arch CT scan was obtained, and 12 implants were planned. For the maxilla, an acrylic tomographic guide with gutta-percha points was fabricated, and planning was performed with 3Shape Implant Studio (3Shape, Copenhagen, Denmark), resulting in a mucosa-supported guide with three fixation points intended for flapless placement (Figure 7).

**Figure 7:**
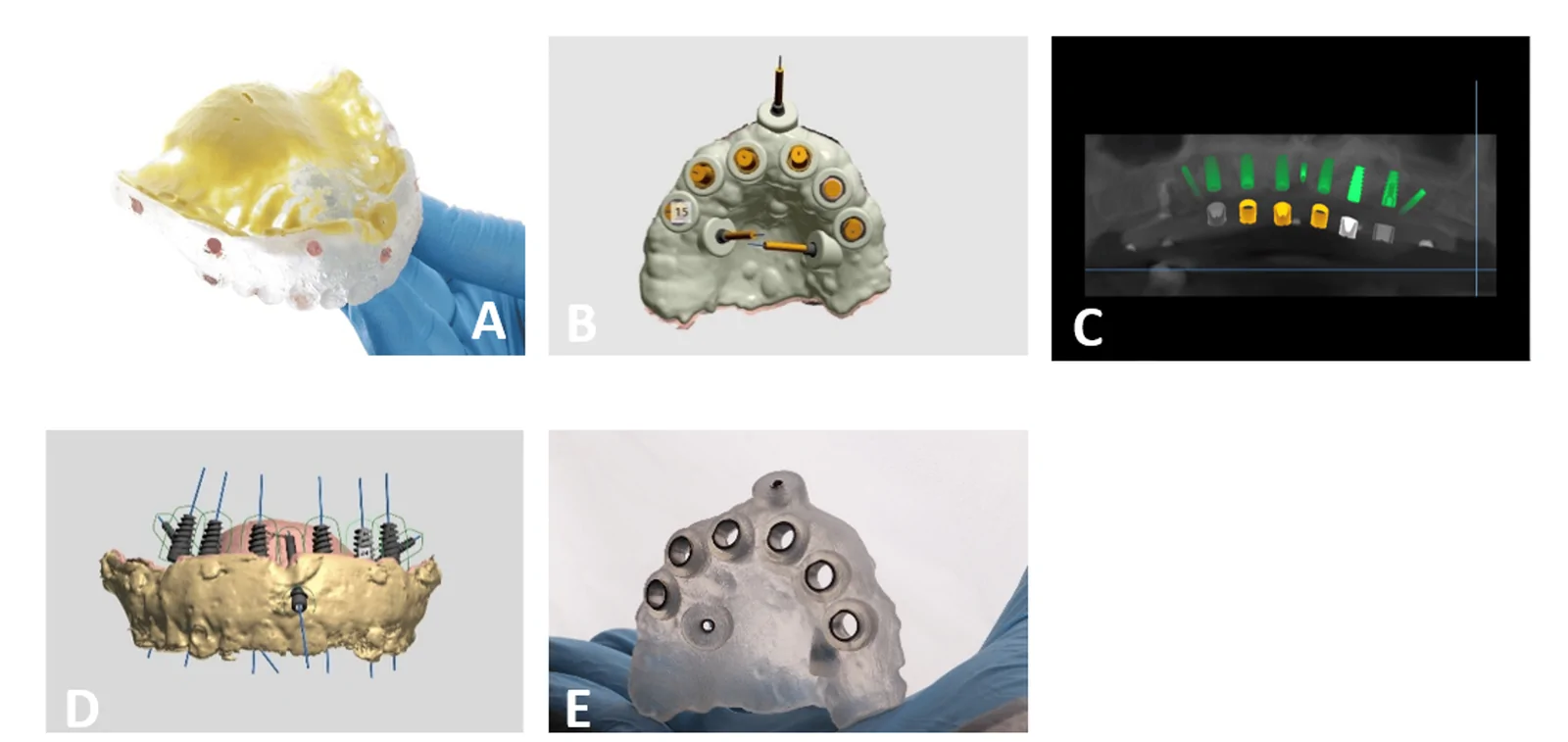
Digital planning of the maxilla. (A) Acrylic tomographic guide with gutta-percha points for tomography and planning in 3Shape Implant Studio (3Shape, Copenhagen, Denmark); (B) virtual design and mucosa-supported guide with three fixation points for flapless surgery; (C) preview of the six upper implants with their sleeves; (D) guide design and occlusal emergence of the implants; (E) printed guide with sleeves in place. The images in this figure were generated with 3Shape Implant Studio (3Shape, Copenhagen, Denmark); the surgical guide was designed in the same software and 3D-printed.

Resin reference points were placed in the mandible for tomography, and an intraoral scan was performed. Planning was carried out with Blue Sky Plan (Blue Sky Bio, Grayslake, Illinois), and two bone-supported guides with three fixation points were designed, one for bone trimming and another for placement (Figure 8) [8,9]. Digital planning specified the position, diameter, and length of each implant, together with safety margins from vital structures. In the maxilla, six implants were planned: positions 16 (4.2 × 11.5 mm), 14 (4.2 × 10 mm), 12 (4.2 × 10 mm), 22 (4.2 × 10 mm), 24 (4.2 × 10 mm), and 26 (4.2 × 10 mm), all placed with a safety distance greater than 2 mm from the maxillary sinus floor and greater than 5 mm from the nasopalatine canal. In the mandible, six implants were planned: positions 36 (5.0 × 10 mm), 34 (4.2 × 10 mm), 32 (3.75 × 10 mm), 42 (3.75 × 10 mm), 44 (4.2 × 10 mm), and 46 (5.0 × 10 mm), all placed with a safety distance greater than 2 mm from the mandibular canal. These parameters were confirmed in the planning software and are displayed in Figures 7, 8.

**Figure 8:**
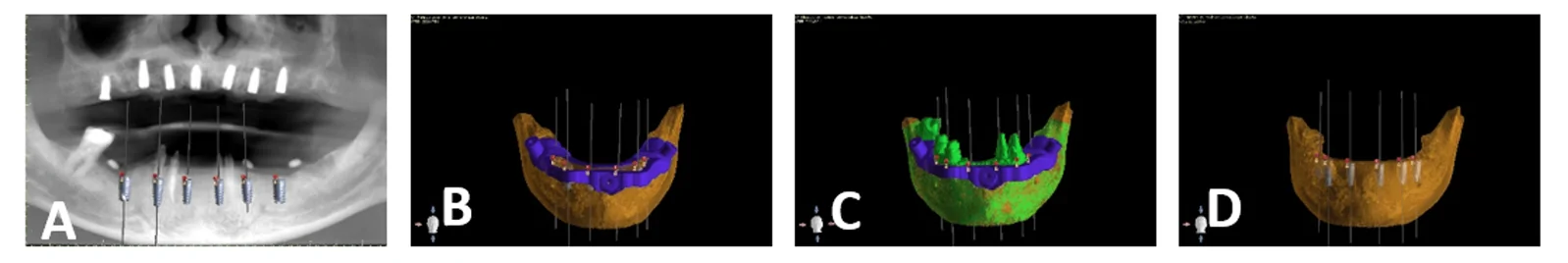
Digital planning of the mandible. (A) Tomography to plan the lower implants in relation to the upper implants; (B) design in Blue Sky Plan of the bone-supported trimming guide; (C) bone-supported placement guide with three fixation points; (D) planned parallelism of the implants.

Implant placement began in the maxilla. Under regional infiltration with 4% articaine and 1:100,000 epinephrine, the mucosa-supported guide was seated and secured with pins; once its stability was confirmed, the soft tissue was removed with a punch at the planned positions. Drilling followed the manufacturer's guided-surgery kit protocol, and six C1 implants (MIS Implants Technologies, Bar Lev, Israel) were placed, all with an insertion torque above 35 N·cm. Cover screws were placed, and the guide was removed without the need for sutures (Figure 9) [8,9].

**Figure 9:**
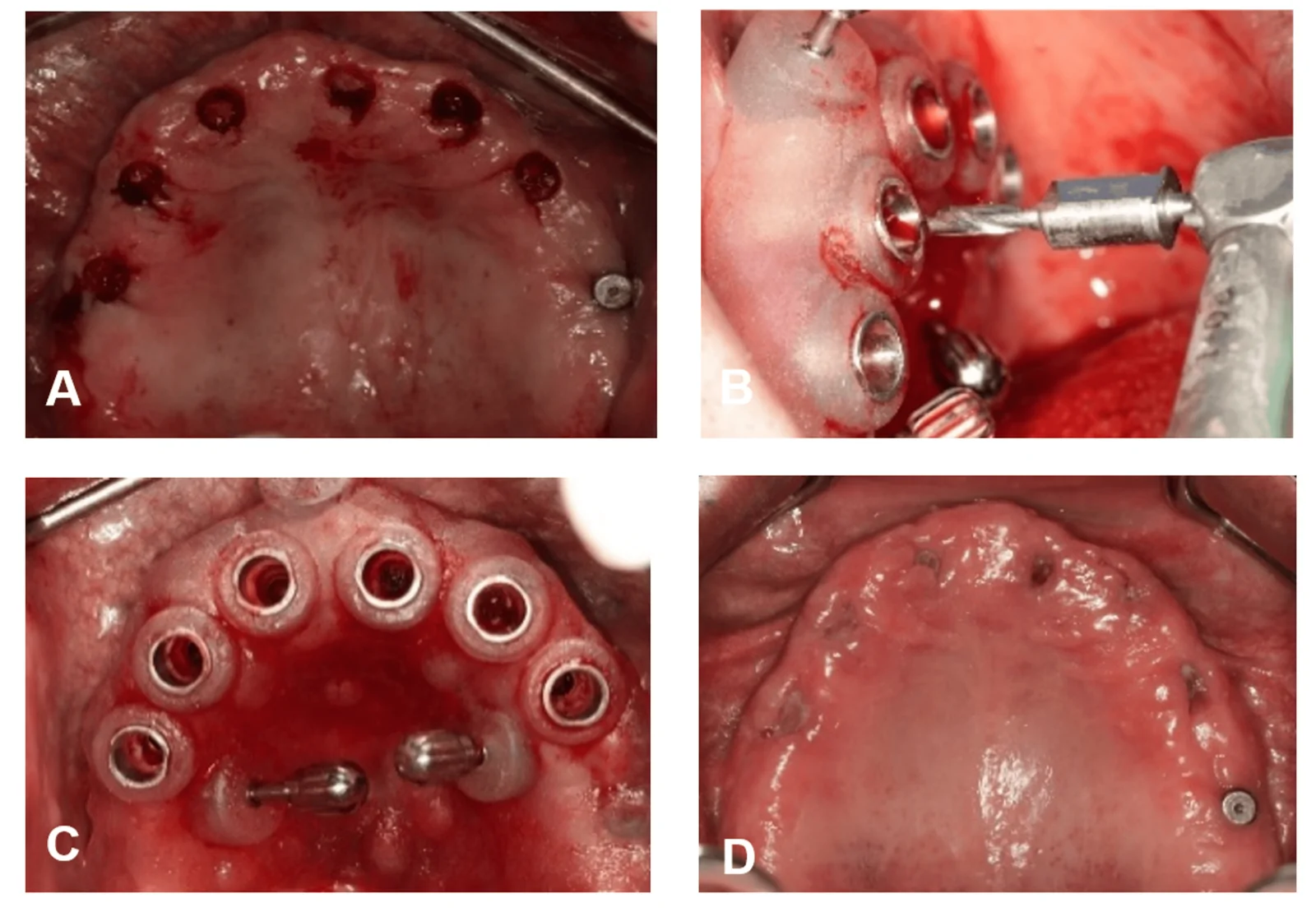
Placement of implants in the maxilla. (A) Removal of mucosa with a punch from the MIS kit; (B) guided drilling according to the manufacturer's protocol; (C) insertion of the six C1 implants with a mucosa-supported guide and torque exceeding 35 N·cm; (D) healing at 7 days.

In the mandible, under a bilateral inferior alveolar nerve block, a full-thickness flap was raised, and teeth 33, 32, 43, 44, and 47 were extracted. The bone-trimming guide was positioned and fixed with pins, and the ridge was regularized with a piezoelectric instrument up to the planned limit. It was then replaced by the placement guide, and six C1 implants were inserted at positions 46, 44, 42, 32, 34, and 36 with their cover screws. At the site corresponding to position 44, plasma rich in growth factors obtained according to the BTI protocol (BTI Biotechnology Institute, Vitoria, Spain) was mixed with autologous bone and covered with an autologous membrane from the same preparation [11,12]. Suturing was performed with 4-0 polytetrafluoroethylene in horizontal mattress stitches, reinforced with simple 5-0 polyglactin sutures (Figure 10). A two-stage healing protocol was used, with cover screws and submerged healing before second-stage surgery.

**Figure 10:**
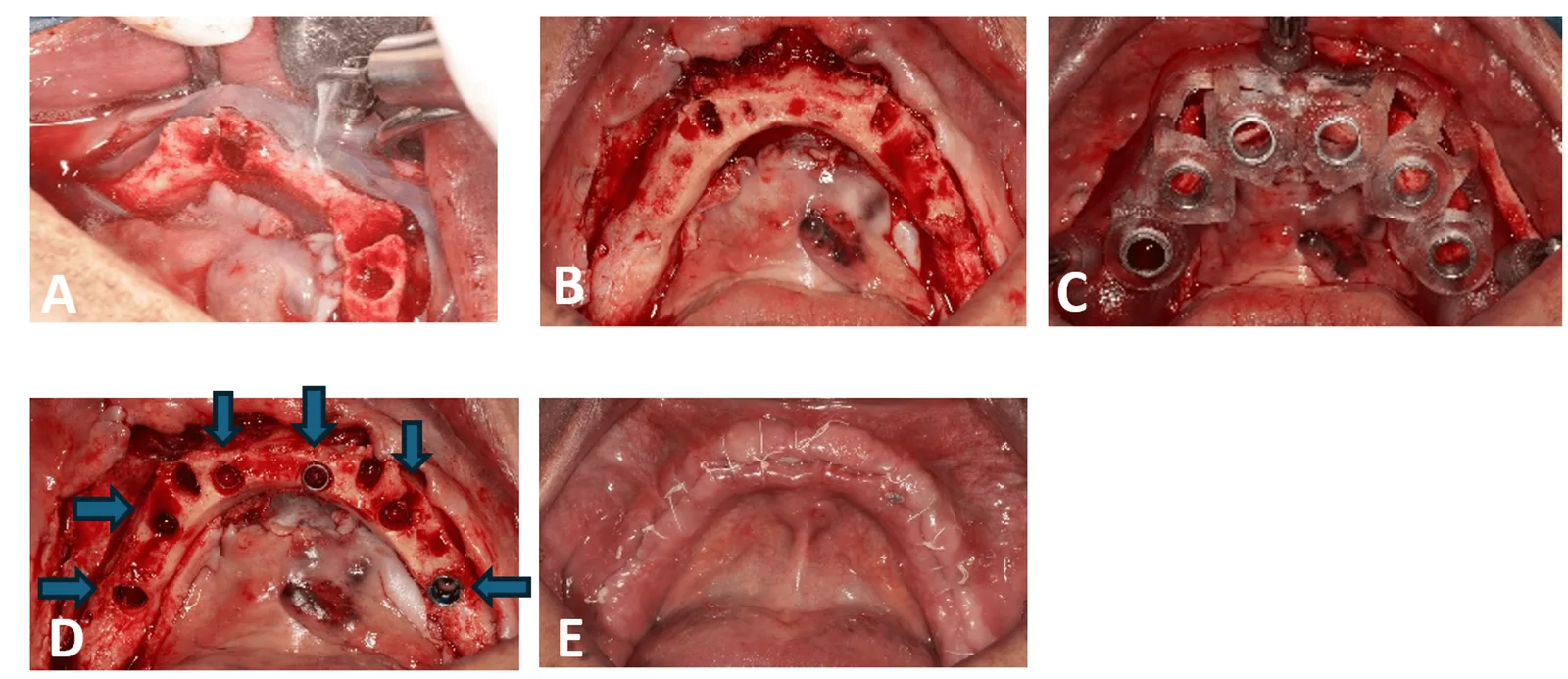
Implant placement in the mandible. (A) Bone-trimming guide and osteotomy with a piezoelectric instrument; (B) result after trimming; (C) placement guide with three fixation pins; (D) insertion of six C1 implants; (E) healing at 15 days.

Four months later, the second surgical phase was performed. In the maxilla, the initial guide was used as a reference to remove the soft tissue with a punch over each implant and to place healing abutments without suturing. In the mandible, using the initial guide as a reference, H-shaped incisions were made to displace the keratinized gingiva toward the buccal aspect, and healing abutments were placed and sutured with simple 5-0 polyglactin sutures at the six positions. Rehabilitation was completed with screw-retained fixed prostheses in both arches (Figure 11). Maintaining an adequate zone and thickness of keratinized mucosa is essential to optimize soft-tissue stability and prevent future peri-implant breakdown [13].

**Figure 11:**
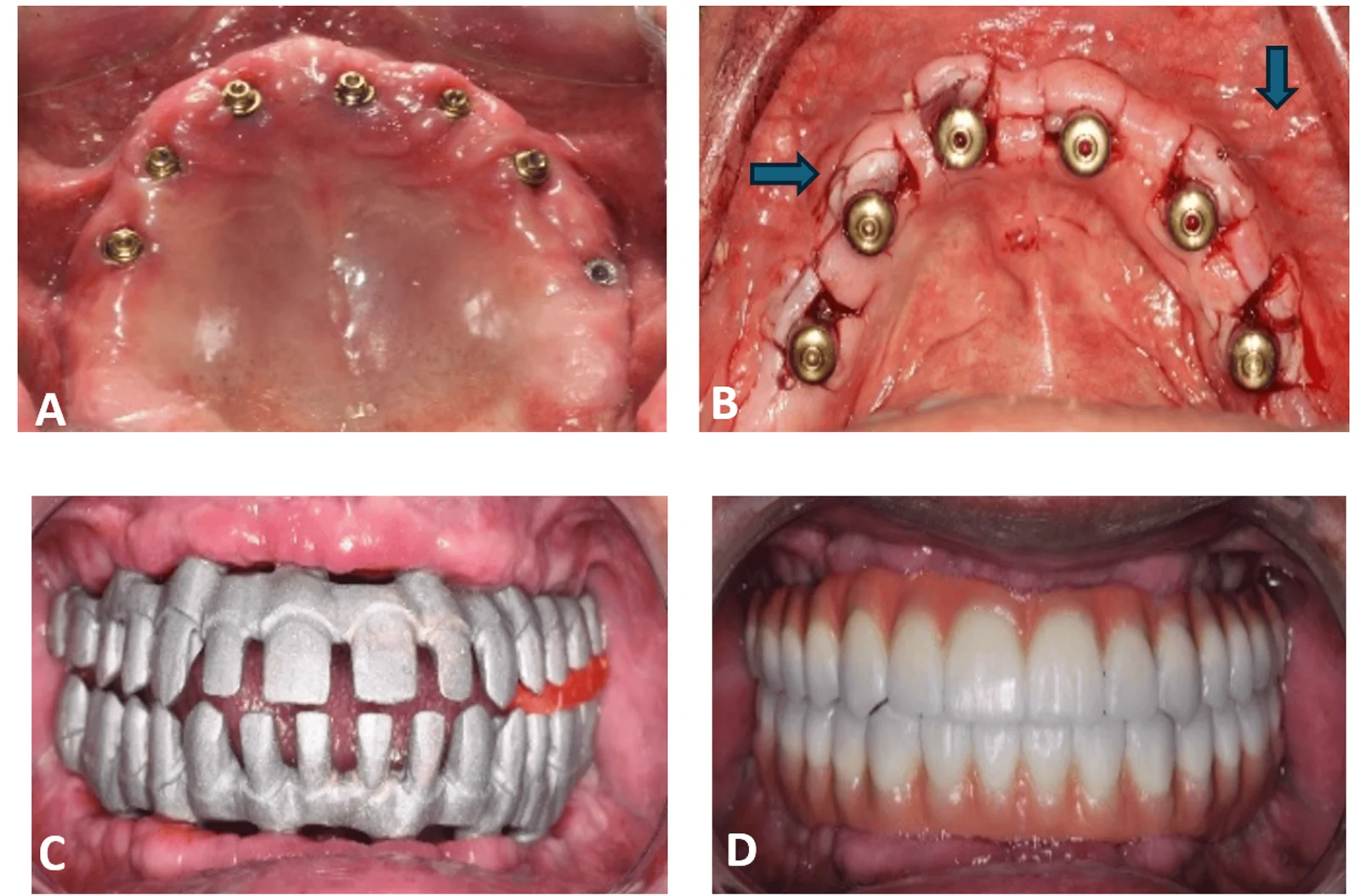
Second surgical phase and rehabilitation. (A) Exposure of the upper implants with a punch and placement of healing abutments without sutures; (B) H-shaped incisions to displace keratinized gingiva and healing abutments in the mandible; (C) framework try-in on both arches; (D) final position of the screw-retained fixed prostheses in the maxilla and mandible.

The follow-up period of 12 months was counted from delivery of the definitive prostheses. Clinical and radiographic records were obtained at the end of active treatment and at the end of the follow-up period. Probing depths remained between 3 and 4 mm at all implants, measured at six sites per implant, with no bleeding on probing at any site at any recall. At each maintenance visit, a six-site protocol was applied for probing depth and bleeding on probing (mesiobuccal, mid-buccal, distobuccal, mesiolingual, mid-lingual, and distolingual). The absence of bleeding was consistently recorded at all sites at every recall appointment, not only at the final 12-month evaluation, confirming sustained peri-implant health throughout the follow-up period. Compared with the reference records, the radiographs showed no evident progression of marginal bone loss; standardized linear measurements were not performed, so this finding is reported descriptively rather than as a quantified value. All implants were surrounded by at least 3 mm of keratinized gingiva (measured at the mid-buccal aspect with a periodontal probe). The retained implant is reported separately from the newly placed implants: at 12 months it showed a probing depth of 3-4 mm without bleeding, consistent with resolution of the treated lesion. The prostheses in both arches remained stable during the observation period, and the patient was able to perform peri-implant hygiene with interdental brushes and irrigators. No biological, mechanical, or prosthetic complications were observed during healing or follow-up. The chronology of the treatment is presented in Table 2.

**Table 2:** Treatment timeline by clinical milestones. Earlier milestones are expressed in months from baseline (Day 0); the final follow-up was performed 12 months after delivery of the definitive prostheses. CBCT: cone beam computed tomography.

Time point	Stage	Procedures performed
Day 0 (baseline)	Diagnosis and phase I	Clinical and CBCT diagnosis; scaling and root planning, oral hygiene reinforcement; diagnostic wax-up and provisionals
Day 0 (surgical phase I)	Explantation, resective therapy, GBR	Explantation of six implants, implantoplasty and decontamination of implant 17, guided bone regeneration of the sites
Month 4	Digital planning and implant placement	Full-mouth CBCT, static guided surgery, placement of 12 implants (torque > 35 N·cm), PRGF at site 44
Month 8	Second-stage surgery	Uncovering of implant, healing abutments; keratinized-tissue management in the mandible
Month 9-10	Prosthetic rehabilitation	Framework try-in and delivery of screw-retained fixed prostheses in both arches
12 months after prosthesis delivery	Final follow-up	PD 3-4 mm at all implants, no bleeding on probing, no evident progression of marginal bone loss, stable prostheses, no complications

## Discussion

In a patient with generalized peri-implantitis, the key is to determine which implants warrant preservation. This decision was based on the morphology and severity of the defect assessed using cone beam computed tomography [3], supplemented by the clinical parameters of each implant; from this, all subsequent actions were derived: one implant underwent resective treatment, while six were removed, followed by reconstruction of the sites [5,6].

The management of advanced peri-implantitis remains debated, and the present approach can be read against that literature. Non-surgical therapy alone is generally insufficient once bone loss is advanced, and surgical therapy (resective, reconstructive, or combined) is usually required [1,4]. Reconstructive therapy can improve radiographic defect fill and clinical parameters at peri-implantitis-related defects, but its outcomes are variable and depend on defect configuration, as shown in the meta-analysis by Tomasi et al. [6]. When the implant itself is considered unsalvageable, several authors favor explantation followed by site reconstruction over attempts to retain a hopeless implant, precisely the rationale followed here [5,10]. Our case differs from most reconstructive series in that it combines, in the same patient, resective therapy for a salvageable implant, explantation with staged regeneration for the hopeless ones, and digitally guided placement of new implants, an integrated sequence that is less frequently reported than each component in isolation.

Monje et al. reported resolution of the disease when implantoplasty was added to debridement and surface decontamination [4]. A subsequent scoping review highlights a reduction in inflammatory signs but also methodological heterogeneity that makes it difficult to compare results between series [14]. In addition, the release of titanium particles during milling and their proinflammatory potential explain why gauze isolation and continuous aspiration were used in this case [15].

Regarding explantation, there is more agreement on when to indicate it than on how to perform it. Monje and Nart emphasize the advisability of anticipating reconstruction and preserving as much bone as possible during the procedure [5]. When burs, trephines, forceps, and piezoelectric instruments are compared, Stajčić et al. reported that the use of a trephine followed by manual extraction is appropriate when bone loss is already extensive [10], which was the case with this patient's mandibular implants.

Defect reconstruction requires precision. A meta-analysis by Tomasi et al. supported reconstructive therapy for defects associated with peri-implantitis [6], whereas a trial by Benic et al. evaluated the regeneration of peri-implant dehiscences using block versus particulate substitutes during implant placement, not delayed reconstruction of an explantation site [7]. For vertical gains, Urban et al. described favorable outcomes with stabilized membranes, albeit with considerable variability among techniques [16].

Plasma rich in growth factors has favorable effects on healing and revascularization, and its combination with autologous bone facilitates graft handling during surgery [11,12]. The published results depend on the harvesting system and protocol used. Because it was applied at a single site and within a broader sequence, its specific contribution in this case cannot be isolated.

One aspect that requires caution is the accuracy of guided surgery. Systematic reviews describe deviations within clinically manageable margins but recommend reserving a safety zone and not taking the complications associated with the procedure for granted [8,9]. Tallarico et al. published a case of computer-guided management of late implant failure from peri-implantitis, with a workflow comparable to that used in this study [17].

The parameters recorded at 12 months, with probing depths of 3 to 4 mm, absence of bleeding, and no evident radiographic progression of marginal bone loss, were consistent with peri-implant health as defined in the current consensus [1]. Monitoring post-restorative probing pocket depth and marginal bone stability over a one-year period serves as a critical benchmark for evaluating peri-implant tissue health and treatment success [18]. The band of keratinized gingiva obtained around the implants also facilitated daily hygiene, a factor that, along with periodic maintenance, weighs more in long-term stability than the technological resource used [1]. Two features of this patient likely contributed and should be acknowledged rather than credited to the surgical protocol alone: his diabetes was well controlled, with a glycated hemoglobin of 6%, and he did not smoke. Because implantoplasty, GBR, guided surgery, and PRGF were applied together within a single sequence and without a comparison group, the specific contribution of each intervention cannot be determined.

This case report has limitations. It is a single case, so its findings cannot be extrapolated and demonstrate clinical feasibility and a favorable short-term outcome only, not effectiveness, predictability, or reproducibility. Peri-implant bone stability was assessed by radiographic comparison without standardized linear measurements; therefore, minor changes could have gone undetected, and the radiographic finding is expressed as an absence of clear progression rather than as a measured value. Long-term stability will also depend on the patient's adherence to recall visits and on home hygiene over time.

## Conclusions

This case illustrates that, in advanced peri-implantitis affecting all of a patient's implants, a staged, prosthetically driven sequence (selective explantation of the hopeless implants, resective treatment of the only recoverable one, guided bone regeneration of the sites, and digitally planned placement of new implants) can restore a functional, screw-retained rehabilitation, with clinical and radiographic parameters consistent with peri-implant health at 12 months after delivery of the definitive prostheses.

As a single, uncontrolled observation in a well-controlled, non-smoking patient, it demonstrates short-term clinical feasibility only; it does not establish the effectiveness, predictability, or reproducibility of the approach, and the contribution of each intervention cannot be determined. Larger, longer-term, multi-patient studies are needed.
